# 3D video-based deformation measurement of the pelvis bone under dynamic cyclic loading

**DOI:** 10.1186/1475-925X-10-60

**Published:** 2011-07-17

**Authors:** Beat Göpfert, Zdzislaw Krol, Marie Freslier, Andreas H Krieg

**Affiliations:** 1 Laboratory of Biomechanics & Biocalorimetry, CM&BE, University of Basel, c/o Bio/Pharmazentrum, Klingelbergstrasse 50-70, 4056 Basel, Switzerland; 2 Paediatric Orthopaedic Department, Children's University Hospital Basel (UKBB) Spitalstrasse 33, 4056 Basel, Switzerland; 3 Laboratory for Movement Analysis Basel, Children's University Hospital Basel (UKBB), Spitalstrasse 33, 4056 Basel, Switzerland

## Abstract

**Background:**

Dynamic three-dimensional (3D) deformation of the pelvic bones is a crucial factor in the successful design and longevity of complex orthopaedic oncological implants. The current solutions are often not very promising for the patient; thus it would be interesting to measure the dynamic 3D-deformation of the whole pelvic bone in order to get a more realistic dataset for a better implant design. Therefore we hypothesis if it would be possible to combine a material testing machine with a 3D video motion capturing system, used in clinical gait analysis, to measure the sub millimetre deformation of a whole pelvis specimen.

**Method:**

A pelvis specimen was placed in a standing position on a material testing machine. Passive reflective markers, traceable by the 3D video motion capturing system, were fixed to the bony surface of the pelvis specimen. While applying a dynamic sinusoidal load the 3D-movement of the markers was recorded by the cameras and afterwards the 3D-deformation of the pelvis specimen was computed. The accuracy of the 3D-movement of the markers was verified with 3D-displacement curve with a step function using a manual driven 3D micro-motion-stage.

**Results:**

The resulting accuracy of the measurement system depended on the number of cameras tracking a marker. The noise level for a marker seen by two cameras was during the stationary phase of the calibration procedure ± 0.036 mm, and ± 0.022 mm if tracked by 6 cameras. The detectable 3D-movement performed by the 3D-micro-motion-stage was smaller than the noise level of the 3D-video motion capturing system. Therefore the limiting factor of the setup was the noise level, which resulted in a measurement accuracy for the dynamic test setup of ± 0.036 mm.

**Conclusion:**

This 3D test setup opens new possibilities in dynamic testing of wide range materials, like anatomical specimens, biomaterials, and its combinations. The resulting 3D-deformation dataset can be used for a better estimation of material characteristics of the underlying structures. This is an important factor in a reliable biomechanical modelling and simulation as well as in a successful design of complex implants.

## Background

Video motion capturing (MoCap) systems are widely used in animation industries and also in biomechanical applications with the main focus of macroscopic gait analysis [1]. Due to systems improvements over the last years, they are now capable of resolutions of 4704 × 3456 pixels (T160, Vicon, Oxford, UK) and sampling rates up to 10000 frames per second (Raptor 4, Motion Analysis Corporation, Santa Rosa, CA, USA). This increased technology opens new application areas where high camera resolution is needed such as in measuring small deformations of biological tissues, dynamic three-dimensional (3D) deformation of bone-implant systems, or to determine movement after fracture fixation [2-6]. Further, micro motion between bone and implant is an important factor in the longevity of a stable bone-implant interface [7-9]. In particular, the characteristics of complex implant systems with bio-absorbable materials or bioactive surface coatings are not completely known [10]. Variation in daily activities may alter the loading conditions in the bone-implant system because of changing material properties on one hand and the influences of operations on the other. However, biomechanical loading tests can never cover the whole variety of different loading conditions during daily activity or consider all the changes over time in the biological or implant structure [11]. Although computation-based modelling tries to include in its calculation process changes of the bone-implant system; its original data must be based on real validated measurements otherwise it can lead to false conclusions [12,13]. Nevertheless local deformation can be measured highly precise with strain gauges [14,15], or linear variable differential transducers [16]. To measure the 3D-deformation of a whole specimen other methods give a better spatial resolution. Therefore the hypothesis was that a three-dimensional video MoCap system has the potential to measure the sub millimetre 3D-deformation of a whole pelvis specimen.

This study describes the application of dynamic 3D-deformation measurement using a 3D video MoCap system to gain data for the development of complex orthopaedic oncological implants around the hip joint of the pelvis including the determination of its accuracy with a 3D micrometre stage. The measurement of 3D-deformation was done by recording the 3D-movement of passive reflective markers glued onto bone surface while applying a dynamic, cyclic, non-constrained, uni-axial load to the pelvic bone. The results will help achieve more stable implant fixation to the bone, which improves the initial conditions for successful osseointegration and therefore support the durability of the implant in a complex environment after a reconstructive surgery for bone tumour.

## Methods

### Determination of the accuracy of the 3D motion-capture system

**Figure 1:**
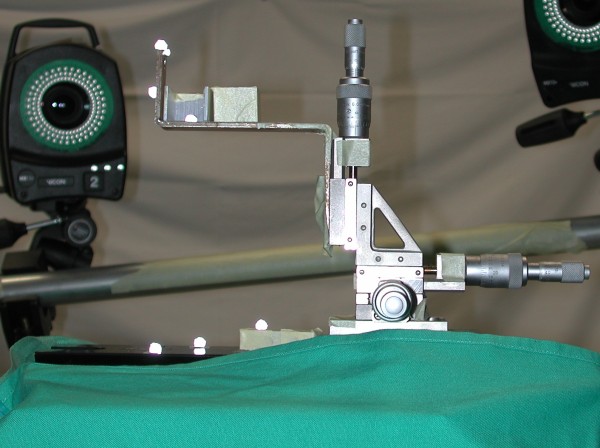
**Accuracy measurement setup**. Setup to determine the accuracy of the 3D motion-capture system.

The accuracy of the measurement setup was determined under the same conditions as the pelvis specimen test was performed. A rigid steel framework equipped with 10 digital high-speed cameras (6 cameras Vicon MX13+, 4 cameras Vicon T40, Vicon, Oxford, UK) was placed around a Servo-hydraulic testing machine (MTS Bionix 858, MTS Eden Prairie, MN, USA). The framework was screwed to the concrete floor and walls of the room to avoid movement of the cameras and have the 10 cameras in the same stable position during measurements. A 3D linear stage (M-461-XYZ-M, Newport Spectra-Physics GmbH, Darmstadt, Germany) equipped with three manually-driven differential micrometres (DM-13, Newport Spectra-Physics GmbH, Resolution 0.0001 mm) was mounted to the table of the Servo-hydraulic testing machine. A Z-shaped steel frame was screwed to the 3D linear stage to hold five dome-shaped, passive reflecting markers with a diameter of 6.5 mm (Prophysics AG, Zürich, CH) (Figure 1). The markers were glued to the Z-shaped frame so that they could be tracked either by 2, 3, 6, or 10 cameras during the accuracy-determination procedure. Three additional markers, visible to all 10 cameras, were placed on to the base of the 3D linear stage as a static reference. The accuracy determination procedure consisted of a 3D-displacement curve with a step function with five steps up and five steps down per axis. Two procedures were performed, each with three independent repetitions. The first procedure had a 0.01 mm displacement in each axis per step and a second of 0.1 mm. The order of the displacement was done by moving the 3D linear stage first in the sagittal (Y), then the transverse (X), and finally in the longitudinal (Z) axes. The new 3D position was then held for a minimum of 5 s before proceeding to the next steps.

Before performing the accuracy determination procedure, the cameras were calibrated as described in the Vicon Handbook using the 120 mm 3-marker calibration wand and the Ergo-Calibration Frame equipped with 9 mm and 9.5 mm markers respectively. The sampling rate of the cameras was set at 60 Hz and the hydraulic pump of the Servo-hydraulic testing machine was running in order to get conditions identical to the 3D-deformation measurements with the pelvic specimen. The recorded 3D video data of the reflecting markers were tracked using the tracking software Vicon Nexus (Vicon, Oxford, UK) without using any filter function and then exported in to Microsoft Excel (Microsoft Corporation, Redmond, WA, USA). The 3D accuracy of the spatial resolution of the each marker was determined during the three middle seconds of the stationary phase after each 3D-displacement step. A simultaneous calculation of the three static markers was done.

### Test procedure with the pelvic specimen

**Figure 2:**
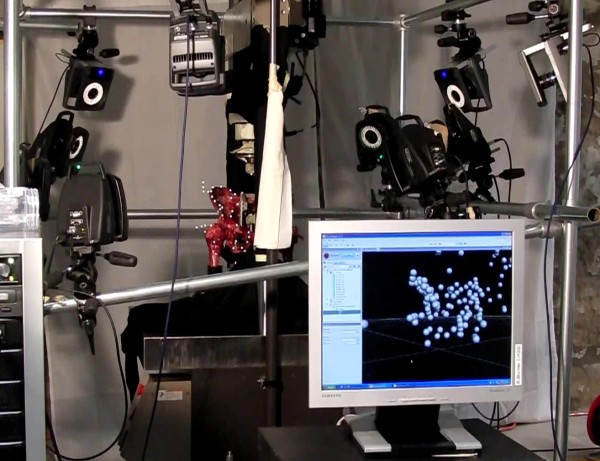
**Test setup**. Setup to measure the dynamic 3D-deformation with a pelvic specimen.

The test object consisted of a complete pelvic specimen including the individual proximal parts of both femora (n = 3). The specimen was fresh-frozen and stored at -25°C. After thawing to room temperature, soft tissues were removed cautiously, leaving the joint capsule, the ventral and dorsal sacroiliac ligaments, the sacrotuberal and sacrospinal ligaments, and the obturator membrane intact. The femora were then fixed with polymethyl methacrylate (PMMA, Beracryl, Swiss-composite, Jegenstorf, CH) in the holding fixture, which was mounted on the table of the Servo-hydraulic testing machine to simulate a two-leg standing position. The load was applied vertically onto the sacrum by the axial actuator of the testing machine with an adjustable fixture at the sacrum, allowing unconstrained rotational and transverse motion [17]. After adjusting the pelvis on the testing machine, 80 reflecting markers (diameter: 6.5 mm, Prophysics AG) were fixed at anatomically defined positions on the bone with cyanoacrylate (Loctide 401; Henkel & Cie AG, Pratteln, CH). Additionally, five quadruple-markers fixed on a T-shaped steel needle were placed at the most lateral point of the iliac crest and the greater trochanter (both sides), and at the proximal spinous tubercles of sacrum. These quadruple-markers were used as a ridge reference system for the different parts of the pelvic specimen during the dynamic loading procedure (Figure 2).

**Figure 3:**
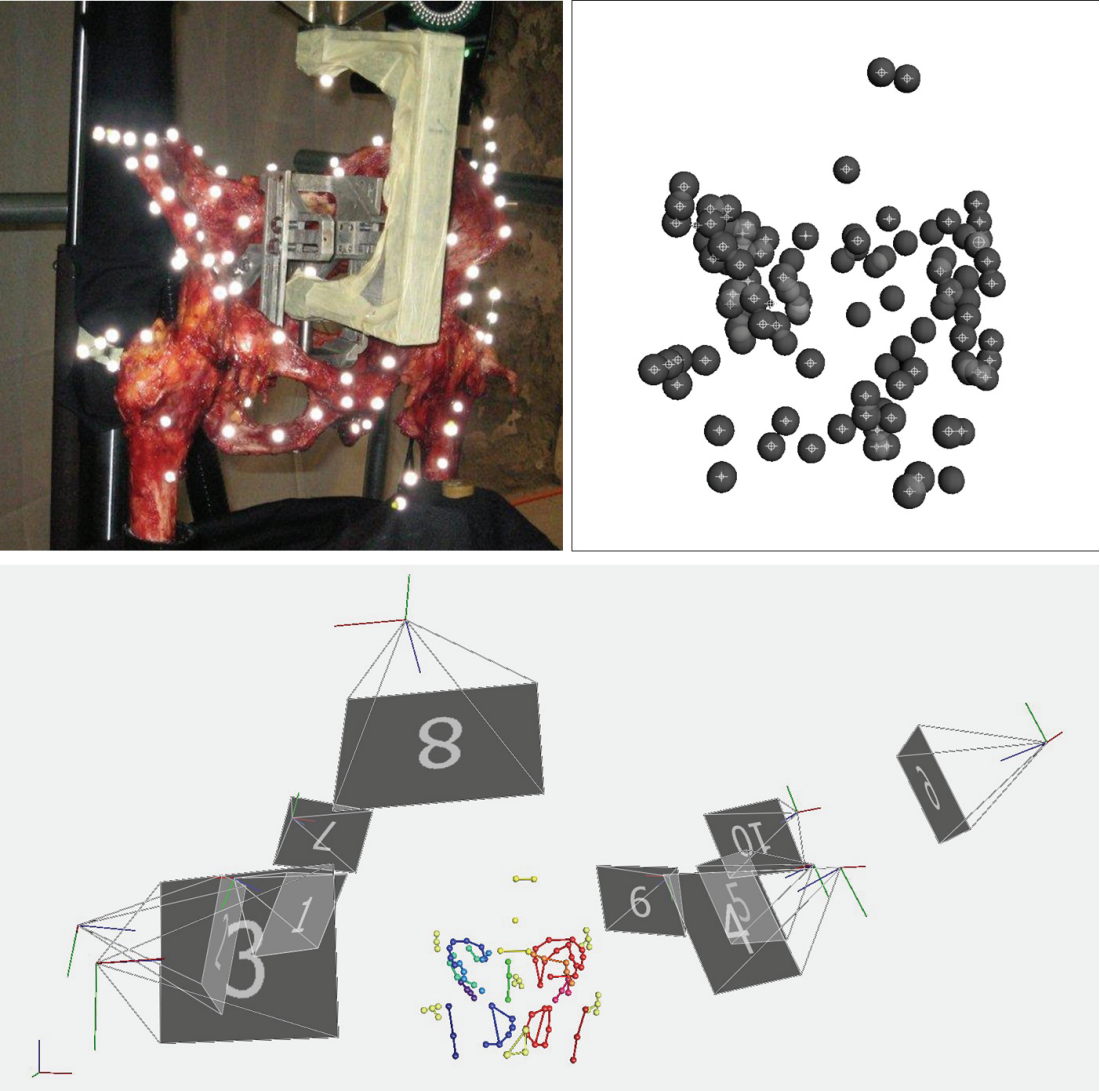
**Data processing steps**. Top left: View of single camera, Top right: View of single Vicon camera before tracking the markers, Bottom: View of the labelled markers at the pelvis with the position of the Vicon-cameras.

The dynamic sinusoidal loading of the pelvic specimen consisted of 100 loading cycles at 1 Hz with amplitudes between 100 N and either 0.5, 1, or 1.5 × body weight. During sinusoidal loading, the 3D-movement of the reflecting markers was recorded synchronously by 10 high-speed digital cameras. The displacement of the axial cylinder and the applied load were recorded simultaneously on the Vicon System at the same sampling rate of 60 Hz (Figure 3).

### Data processing of the 3D marker data of pelvic specimen

The recorded 3D video data of the reflecting markers were reconstructed and tracked using the Vicon Nexus software without using any filter function. Each tracked marker was seen simultaneously by a minimum of 3 cameras. The dataset of each loading condition consists of the global 3D coordinates of all the markers and the applied load. It was exported into a CSV file for further use like in graphical animation software or displacement computation.

Analysis of the 3D-displacement of markers was done using Microsoft Excel. Calculation of the 3D distance change between two markers was done using the following equation:

Where Δd(t) is the temporal distance between the different spatial coordinates (x_i_(t); y_i_(t); z_i_(t)) of two markers over the loading cycles.

## Results

### Accuracy of the 3D motion-capture system setup

The resulting accuracy of the system depended on the number of cameras tracking a marker, which is represented by the magnitude of spatial movements for a stationary marker and is equal to the noise level. The noise level for a marker tracked by two cameras during the stationary phase of the calibration procedure was ± 0.036 mm. The noise level of a tracked marker decreased if it was seen by more cameras, and reached nearly the same level of ± 0.022 mm with 6 cameras as that seen with 10 cameras. The noise level of the 3D position of the static markers tracked by all 10 cameras was ± 0.020 mm (Table 1).

**Table 1:** Accuracy of the measurement setup depending on the number of cameras tracking a marker

Marker visible by n cameras	2	3	6	10	10 static
Noise level [+/- mm] at 0.01 mm step procedure	0.036	0.029	0.021	0.021	0.020

Noise level [+/- mm] at 0.1 mm step procedure	0.035	0.031	0.022	0.018	0.016

**Figure 4:**
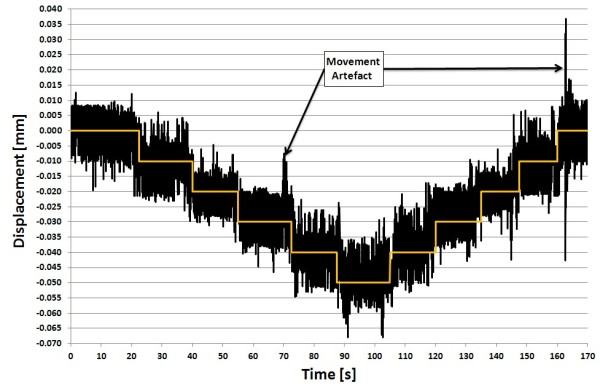
**Comparison: Z-set-value vs. measured z value**. Comparison of the Z-set-value (white line) to the measured z value (black line) tracked by four cameras. Movement artefact occurred due to touching of the framework with the cameras, while moving the 3D linear stage.

The noise level for a marker seen even with 10 cameras was bigger than the detectable movement of 0.010 mm obtained with the 3D linear stage with a manually movement resolution of 0.0001 mm. Therefore the limiting factor was the noise level, which resulted in a measurement accuracy for the dynamic test setup of ± 0.036 mm for a marker tracked by two cameras. Herewith the hypothesis could be fulfilled, that 3D-measurements in the sub millimetre range with this 3D video MoCap-system were possible (Figure 4).

### Examples of 3D-displacement and 3D-deformation between two markers

To illustrate the movement of the specimen and deformation of the pelvis, two pairs of markers were analysed. One pair was chosen at the iliac crest and another between the acetabulum and femur. The loading conditions were the same; a sinusoidal loading of 100 cycles at 1 Hz with a loading amplitude between 100 N and 900 N (1 × body weight). The 10^th ^and 90^th ^cycles were analysed.

**Figure 5:**
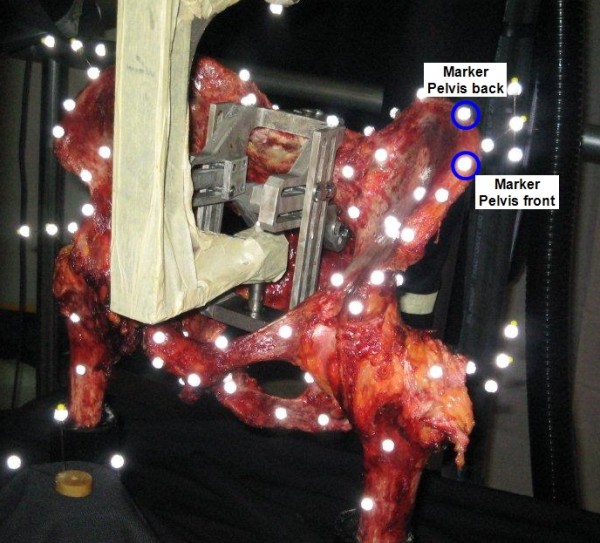
**Markers at the iliac crest**. Markers at the iliac crest to determine the 3D-displacement and 3D-deformation.

**Figure 6:**
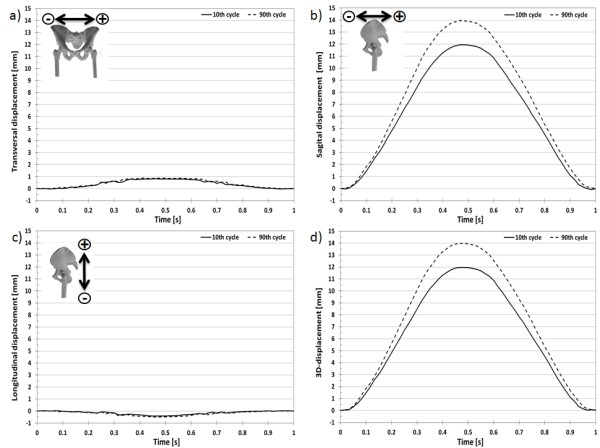
**3D-displacement of the front pelvis marker at the iliac crest**. 3D-displacement of the front pelvis marker at the iliac crest at the 10^th ^and 90^th ^loading cycle with a sinusoidal load between 100N and 900N.

**Figure 7:**
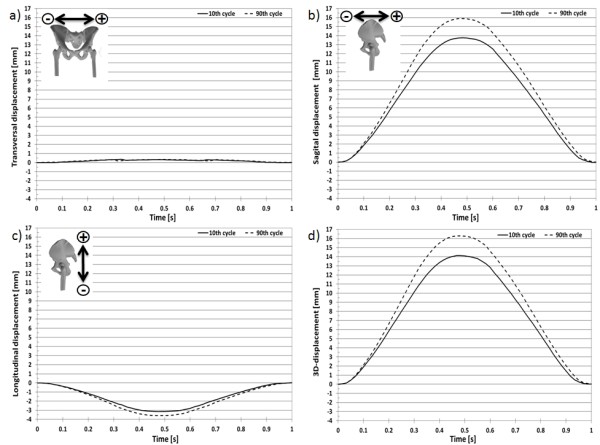
**3D-displacement of the back pelvis marker at the iliac crest**. 3D-displacement of the back pelvis marker at the iliac crest at the 10^th ^and 90^th ^loading cycle with a sinusoidal load between 100N and 900N.

**Figure 8:**
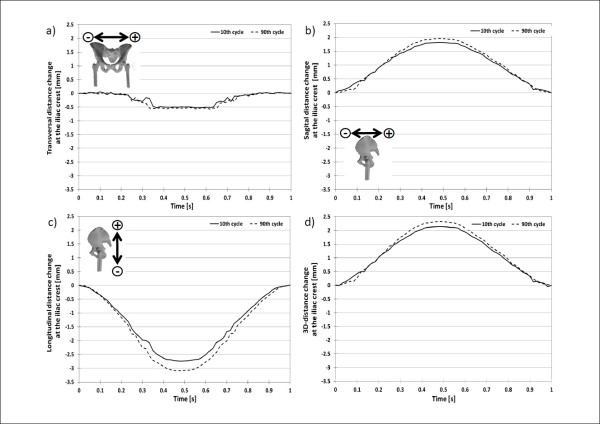
**3D-deformation at the iliac crest**. 3D-deformation between the two markers at the iliac crest at the 10^th ^and 90^th ^loading cycle with a sinusoidal load between 100N and 900N.

At the iliac crest (Figure 5), the total 3D-displacement between a load of 100N and 900N for the pelvis front marker at the 10^th ^and 90^th ^cycles was 11.972 ± 0.036 mm (Figure 6) and 13.971 ± 0.036 mm (Figure 7), respectively. The total displacement of the pelvis back marker was a little bit bigger with 14.188 ± 0.036 mm and 16.294 ± 0.036 mm at the 10^th ^and 90^th ^cycles, respectively for the same loading condition. The 3D-deformation of the iliac crest for the 10^th ^and 90^th ^cycle was 2.146 ± 0.072 mm and 2.325 ± 0.072 mm, respectively. The difference of the deformation between the two loading cycles was 0.179 ± 0.072 mm (Figure 8).

**Figure 9:**
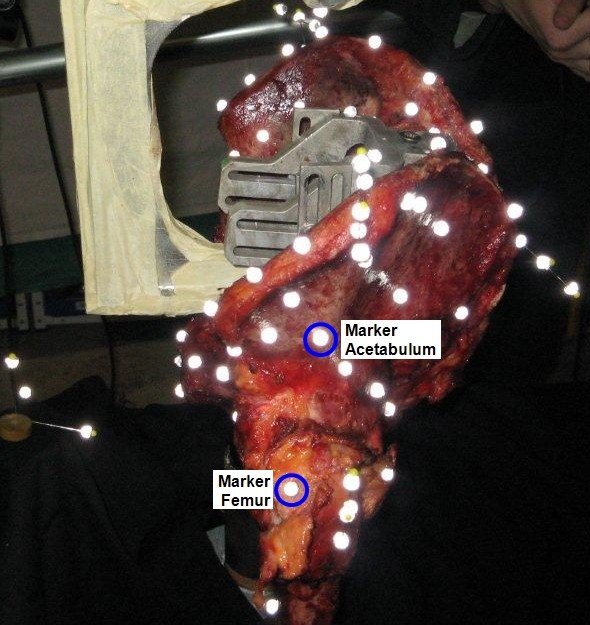
**Markers around the hip joint**. Markers at the acetabulum and femur to determine their 3D-displacement and 3D-deformation.

**Figure 10:**
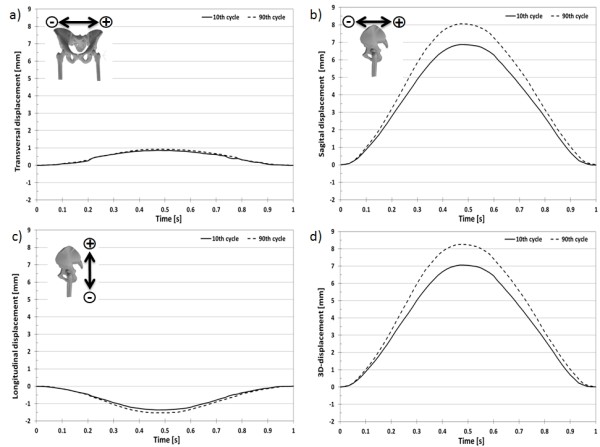
**3D-displacement of the acetabulum marker**. 3D-displacement of the marker at the acetabulum at the 10^th ^and 90^th ^loading cycle with a sinusoidal load between 100N and 900N.

**Figure 11:**
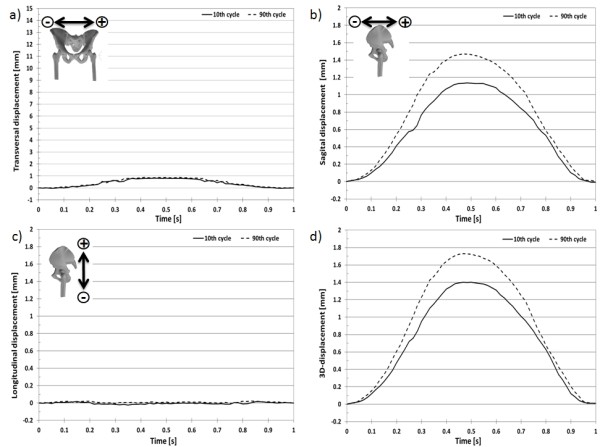
**3D-displacement of the femur marker**. 3D-displacement of the marker at the femur at the 10^th ^and 90^th ^loading cycle with a sinusoidal load between 100N and 900N.

**Figure 12:**
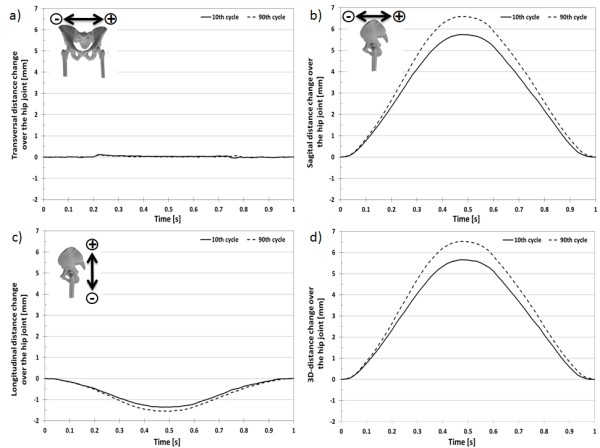
**3D-deformation around the hip joint**. 3D-movement between the two markers at the acetabulum and femur at the 10^th ^and 90^th ^loading cycle with a sinusoidal load between 100N and 900N.

The 3D-displacement for the selected marker at the acetabulum (Figure 9) over a loading cycle at the 10^th ^and 90^th ^cycles was 7.055 ± 0.036 mm (Figure 10) and 8.255 ± 0.036 mm (Figure 11), respectively. The 3D-displacement at the femur was much smaller; being 1.402 ± 0.036 mm and 1.730 ± 0.036 mm at the 10^th ^and 90^th ^cycles, respectively. The 3D-distance change between the two markers across the hip joint at the 10^th ^and the 90^th ^cycle was 5.656 ± 0.072 mm and 6.526 ± 0.072 mm, respectively. The difference in the 3D-distance change between the two loading cycles was 0.871 ± 0.072 mm (Figure 12).

## Discussion

The chosen system, combining 3D-video MoCap with servo-hydraulic material testing, allows dynamic 3D-displacement measurement of the reflecting markers placed on the surface of a specimen in the sub millimetre range. The accuracy of the measurable marker displacement with a 3D video MoCap system depends on the number of cameras tracking a single marker simultaneously, on the size and shape of the markers, and the quality of the camera (CCD-Chip, objective, camera fixation). The accuracy of ± 0.036 mm reached with the setup used in this study is in the range published by Windolf et al. [18], or by Lujan [19]. The setup used by Liu et al. [3] has a higher accuracy but also a smaller measurement volume. The accuracy could be increased by more cameras tracking simultaneous each markers, cameras with a higher resolution and a lower noise level or with bigger markers [18].

The advantage of bigger markers is that there is better reflection of the camera light source at the reflecting marker surface towards the detecting CCD-chip due to smaller curvature of the surface [18]. On the other hand, bigger markers lead to bigger inter-marker distance, or increase the risk of covering some markers due to the bigger volume. A bigger marker distance or less visible markers would lead to a reduction in resolution of the deformation measurement. However, smaller markers could increase the resolution of the deformation due to the smaller inter-marker distance but would be more difficult to handle and place on the surface of the specimen. Additionally a too small the inter-marker distance increases also the risk that two markers merge into one reflecting spot. In this case, a marker would not be properly tracked and its 3D data would be lost. Therefore the dome-shaped markers with a diameter of 6.5 mm, and, based on experience, an inter-marker distance of at least two times the marker diameter are a good compromise between high accuracy and easy handling needed for gluing onto the bony surface.

It has to be taken in account that the marker displacement is usually a combination of the global movement of the specimen in space and deformation occurring through the loading process. However, the movement path of the markers during a loading cycle comprises all the information about the whole specimen and the local loading conditions. Nevertheless, it is possible to draw some conclusions about the bony structure of the specimen or the movement of a joint between different markers, without knowing the exact underline bony structure. Although the quality of the information behind the surface displacement data will be improved by additional non-destructive technologies like computed tomography (CT) scans, further improvements might be possible by local micro-CT scans [20].

A big benefit of the used plastic reflecting marker is that they don't induce any artifacts on the CT-images. Therefore it is relative easy to combine the 3D-deformation data with the CT-based bone-density and structure information. The spatial resolution (voxel size) of the current CT-scanner is in the range of 1 mm. That means the CT-scanner has about 20-times smaller resolution than the MoCap-system. However, this difference in resolution is not the limiting factor by combining the MoCap-system and the CT-scanner. The limiting factor to acquire a high precision resolution 3D bone deformation in connection with the CT-data of the underlining bony structure is actually the inter-marker distance of the reflecting markers as described above.

## Conclusions

The combination of 3D video MoCap, and material testing opens new possibilities in dynamic testing. Combined with CT-data of the underlining bony structure, it becomes highly valuable framework for finite element modelling of complex implants [13]. It may also improve the development process of new implant technologies through better biomechanical compatibility with the patient specific musculoskeletal anatomy.

## Competing interests

The authors declare that they have no competing interests.

## Authors' contributions

AHK, ZK and BG planned the study, BG and MF analyzed the data, AHK and BG wrote the first draft of the manuscript. AHK, MF, ZK and BG took care of revisions. BG, ZK and AHK contributed to interpretation of the results and writing of the manuscript. All authors have read and approved the final manuscript.
